# Egg quality characteristics of South African Potchefstroom Koekoek and commercial Lohmann brown layer chickens: A comparative study

**DOI:** 10.1371/journal.pone.0340483

**Published:** 2026-01-12

**Authors:** Victoria Rankotsane Hlokoe, Thobela Louis Tyasi, Vusi Gordon Mbazima

**Affiliations:** 1 Department of Agricultural Economics & Animal Production, School of Agricultural & Environmental Sciences, University of Limpopo, Limpopo, South Africa; 2 Department of Biochemistry, Microbiology & Biotechnology, School of Molecular and Life Sciences, University of Limpopo, Limpopo, South Africa; Ain Shams University Faculty of Agriculture, EGYPT

## Abstract

Egg quality is an important feature in egg production and is affected by many factors, including the genetic makeup of the bird. However, the influence of the chicken genotype on egg quality traits is limited and inconclusive. Therefore, this study aimed to highlight the influence of genotype on the external and internal egg quality traits of the Potchefstroom Koekoek and Lohmann Brown layers. A total of 600 eggs (300 eggs per genotype) was used, and a cross-sectional experimental design was used. The findings displayed that all external egg quality traits were significantly (P < 0.05) affected by the genotype, except for the unit shell surface weight (P ≥ 0.05). The outcomes also displayed that the genotype significantly (P < 0.05) affected all internal egg quality traits. The most affected traits favored the Lohmann Brown over the Potchefstroom Koekoek chickens. In conclusion, the outcomes showed significant differences in the external egg quality traits such as the egg length, shell weight, eggshell index, egg width and shell ratio, and the significant differences in the internal egg quality traits such as yolk/ albumen, yolk weight, albumen weight, albumen ratio and yolk ratio between the Potchefstroom Koekoek and Lohmann Brown chickens.

## Introduction

Eggs contain nutrients for developing embryos and provide proteins for human beings [1]. The demand for eggs continues to increase worldwide with an increasing human population [2]. Therefore, improvement of egg production in chickens is required to meet consumer demand. Egg weight is an economically important trait that requires careful attention from farmers, as it affects pricing [3]. Egg weight is affected by several egg quality traits including egg width, length, shell weight, ratio, yolk and albumen weight, and yolk ratio [4].

It has been found that native chickens produce more delicious and high-quality eggs and meat than high-producing hybrids, which are not well suited to the local environment [5]. Furthermore, indigenous breeds with less selection pressure continue to provide high-quality products from extensive rearing systems that are becoming more and more acceptable to consumers [6], while commercial hen breeds that are selected over an extended period of time based on the quantity of raw materials produced (meat or eggs) have seen a decline in quality [7]. Prioritizing local breed birds is beneficial for both preserving priceless genetic resources and supplying high-quality products [7]. Commercial Lohmann Brown Chickens cannot survive better under unfavorable conditions, including scavenging conditions, and are less hardy [8]. However, an indigenous Potchefstroom Koekoek chicken can survive well under unfavorable environmental conditions, including scavenging for food; it is hardy and disease-resistant [9–11]. Nonetheless, the egg production of these chickens is low, which results in reduced egg yield, which in turn affects the profitability of farmers [12].

Studies have been conducted on the influence of breed on egg quality traits in the Cairo L-2 strain, a local layer strain and commercial Lohmann Brown-Lite (LBL) strain [13], Oravka and Rhode Island Red laying hens [14] and Rhode Island Red and Baledy chicken breeds [15] and in Polbar and Greenleg Partridge hens [16]. However, there are no studies comparing the egg quality traits of the Potchefstroom Koekoek and Lohmann Brown chickens. Thus, the study’s objective was to evaluate the effect of the genotype on the external and internal egg quality traits of the Potchefstroom Koekoek and Lohmann Brown chickens. The study hypothesized that the breed does not have an influence on the external and internal egg quality traits of Potchefstroom Koekoek and Lohmann Brown chickens. The findings of this study will help South African Potchefstroom Koekoek chicken farmers improve egg characteristics compared with the egg characteristics of the commercial Lohman Brown strain.

## Materials and methods

### Ethical approval

University of Limpopo Animal Research and Ethics Committee provided ethical approval (AREC/55/2023: PG) prior to commencement of the current study.

### Study site

This study was conducted at the University of Limpopo Experimental Farm in South Africa. Temperature ranges, coordinates, and rainfall patterns were similar to those explained by Shabalala et al. [17]. In short, summertime temperatures (November to January) range from 20 to 36°C, whereas wintertime temperatures (May to July) range from 5 to 25°C. Longitude 29°44’07.4“E and latitude 23°53’23.7”S are the coordinates of the University of Limpopo. It receives lower than 400 mm of rainfall on average per year.

### Experimental animals and management

A total of 100 Potchefstroom Koekoek and 100 Lohmann Brown chickens were used in this study. The chickens were purchased at 18 weeks from Bosveld farm in Bela Bela, and the diets were acquired from Polokwane, Driehoek feeds in South Africa. These hens were housed in an intensive production system under the same environmental conditions. The chicken management was done as explained by Hlokoe et al. [11]. The chickens were fed the same diets, which included a standard ration of 20% CP and 2800 Kcal/kg ME during the first eight weeks, and 16% CP and 2800 Kcal/kg ME during the growing stage (9–18 weeks). The chickens had unrestricted access to water and were fed laying mash from Diehoek Feeds in South Africa. The following nutritional components made up the diet: metabolizable energy (2453.60 Kcalkg-1), crude protein (16%), crude fats and oils (4.3%), crude fibres (4.8%), crude ash (13.6%), calcium (4.3%), phosphorus (0.6%), sodium (0.15%), lysine (0.7%), and methionine (0.35%). The NRC declared that the diet was balanced (1994). Birds were vaccinated against Marek’s and Newcastle at day 1, Gumboro at 7 days, typhoid at 6 weeks and fowl pox at 12 weeks.

### Egg collection

A total of 600 eggs (300 eggs per breed) were randomly collected from Potchefstroom Koekoek and Lohmann Brown chickens at the age of 24 weeks for 3 weeks to assess the egg quality traits. The sample size of this study determined following the study of Saroj et al. [18] which used 154 eggs. Every Monday and Thursday, in the morning and evening, eggs were gathered. In order to assess the internal and external egg quality characteristics, the eggs gathered were sent at room temperature to the lab. Every Monday and Thursday after collection, the egg quality traits were measured to avoid long storage periods. Lohman Brown chickens started laying at 18 weeks, while Potchefstroom Koekoek started laying eggs at 20 weeks of age. However, the eggs were collected at 24 weeks for the study.

### Measurement of external and internal egg quality traits

The external egg quality traits that were collected included egg length (EL), shell weight (SW), eggshell index (ESI), shell ratio (SR), egg width (EWD) and unit surface shell weight (USSW). Internal egg quality traits involve the yolk/ albumen (Y/A), yolk weight (YW), albumen weight (AW), albumen ratio (AR) and yolk ratio (YR). Briefly, the egg weight and SW were determined using a weighing scale (Medidata®, USA) calibrated in grams (g), with an accuracy of 0.01 g, while EWD and EL were taken using a Vernier caliper (Mitutoyo®, Japan) calibrated in mm, with an accuracy of 0.01 mm. To measure the yolk and albumen weight, the individual eggs will be carefully broken out to avoid breaking the sheaths that surround the albumen and yolk. The egg yolk was separated from the albumen using egg yolk separator, and an electronic weighing scale was used to measure the egg yolk’s weight. The weight of the yolk and shell was deducted from the weight of the entire egg to determine the albumen weight. Using formulas outlined by Markos et al. [19] and Hlokoe et al. [11], additional internal and external egg quality traits were computed.


$$\text{Shape index }(\%)=\;\frac{\text{egg width}}{\text{egg length}}\text{ x 100}$$

$$\text{Unit surface shell weight }(\text{g}/{\text{cm}}^{\text{2}})\;=\;\frac{\text{shell weight}}{\text{shell surface area}}$$

$$\text{Shell ratio }(\%)=\;\frac{\text{shell weight}}{\text{egg weight}}\text{ x 100}$$

Albumen weight (g) = egg weight − (yolk weight + shell weight)

$$\text{Albumen ratio }(\%)=\;\frac{\text{albumen weight}}{\text{egg weight}}\text{ x100}$$

$$\text{Yolk ratio }(\%)=\;\frac{\text{yolk weight}}{\text{egg weight}}\text{x 100}$$

$$\text{Yolk}/\text{albumen }=\;\frac{\text{yolk weight}}{\text{albumen weight}}\text{ x 100}$$

### Statistical analysis

The data analysis was conducted using version 28.0 of the Statistical Package for Social Sciences (IBM SPSS) [20]. For the egg weights, descriptive statistics were computed, and Pearson’s correlation was used to look at the relationship between the traits. The influence of breed on egg quality traits was determined using Student’s t-test. A p-value of 0.05 was used to test significant differences between chicken breeds. The effect of breed was computed using the following equation:


$${\text{Y}}_{\text{ij}}\;=\;\mu \;+\;{\text{S}}_{\text{i}}\;+\;{\text{e}}_{\text{ij}}$$

Where, Y_ij_: The j^th^ observation of the i^th^ genotype.

μ: The overall mean.

Si: The fixed effect of the i^th^ genotype.

e_ij_: Residual error

## Results and discussion

### Descriptive statistics of egg weight

**Fig 1:**
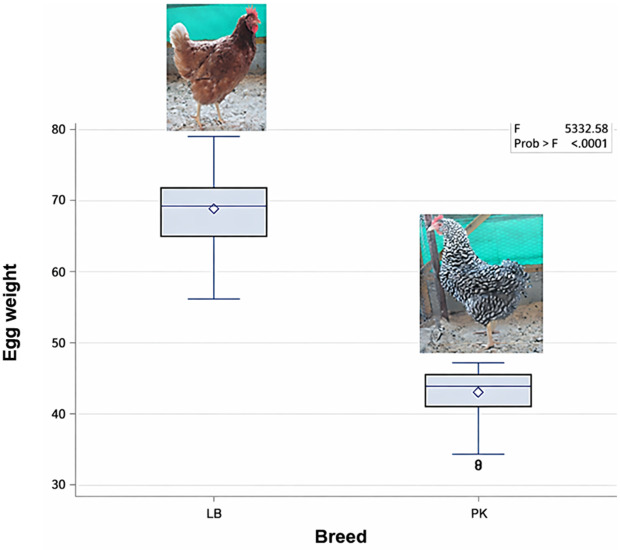
Boxplot showing the minimum, maximum, median, 25th quartile, and 75th quartile values of egg weight. LB = Lohmann Brown chicken strain, PK = Potchefstroom Koekoek chicken strain.

A summary of egg weight of the Potchefstroom Koekoek chicken breed and Lohmann Brown strain is displayed in Fig 1. The findings showed that the egg weight of Lohmann Brown chickens was higher than that of Potchefstroom Koekoek chickens. Furthermore, the boxplot shows the minimum, 25th percentile (first quartile), median, 75^th^ percentile (third quartile), and maximum egg weight values for both chickens. g, the Lohmann Brown shows a minimum value of 55.84 g, first quartile and median which is greater than 55.84 g, a third quartile that is greater than 70 g and a maximum of less than 80 g. The boxplot of Potchefstroom Koekoek showed a minimum of 32.05 g, first quartile and median of greater than 32.05 g, a third quartile of higher than 40 g and a maximum of lower than 50 g.

### Effect of genotype on external egg quality traits

The effects of genotype on the external egg quality traits are shown in Table 1. The results showed that all external egg quality traits were significantly (P < 0.05) affected by the genotype, except for the unit shell surface weight (P < 0.05). The findings further revealed that the egg weight was significantly (P < 0.5) affected by the genotype, with Lohmann Brown having a higher mean numerical value than the Potchefstroom Koekoek chicken breed. The results also showed that the genotype significantly (P < 0.5) influenced egg length, egg width, shell weight, shape index, and shell ratio, whereas Potchefstroom Koekoek performed better in terms of shell ratio and shape index than the Lohmann Brown chicken strain. Egg weight varies among different chicken breeds and is influenced by egg characteristics [4]. Oleforuh-Okoleh [21] reported similar results in Black Olympia and Nigerian local chickens, which showed that the breed influenced egg weight, shell weight, and shell index. Hanusová et al. [14] reported significant differences in external egg quality traits such as the egg length and egg width in dual-purpose laying hens of the Oravka and Rhode Island Red breeds. Eggs of the Oravka breed were heavier than those of the Rhode Island Red breed, and this may be due to genetic and feed effects. Tyasi et al. [22] reported that the breed influenced egg quality traits such as egg width, egg length and shell weight, with Hy-line Silver Brown chicken breed performing better than Potchefstroom Koekoek chicken breed. This might be due to genetic differences and environmental factors such as feeds. This study suggests that the egg weight and egg quality traits of the Potchefstroom Koekoek chicken breed might require genetic improvement for improved profitability and consumer acceptability, as this breed possesses good adaptability and disease resistance traits, which makes it less costly to rear. Egg quality traits influence consumer acceptability, which in turn affects the profits of farmers [1].

**Table 1:** Mean ± SE of external egg quality traits affected by genotype.

Egg quality traits	Potchefstroom Koekoek	Lohmann Brown	Probability Value
Egg weight (g)	42.57^b^ ± 3.13	68.30^a^ ± 5.23	< 0.001
Egg length (mm)	52.12^b^ ± 1.99	58.32^a^ ± 2.37	< 0.001
Egg width (mm)	40.90^b^ ± 0.84	45.07^a^ ± 1.28	< 0.001
Shell weight (g)	6.32^b^ ± 0.75	9.19^a^ ± 1.11	< 0.001
Shape index (%)	78.58^a^ ± 3.40	77.40^b^ ± 3.54	< 0.001
Unit shell surface weight (g/cm^2^)	0.10 ± 0.01	0.10 ± 0.01	0.77
Shell ratio (%)	14.93^a^ ± 2.17	13.52^b^ ± 1.88	< 0.001

SE: standard error; a, b means in the same row with different superscripts are significantly different (P < 0.05).

### Effect of genotype on internal egg quality traits

Table 2 shows the effects of genotype on internal egg quality traits. The results revealed that genotype significantly (P < 0.05) affected all internal egg quality traits. Lohmann Brown had the highest yolk weight, albumen weight, and albumen ratio compared to the Potchefstroom Koekoek chicken breed. The results also showed that Potchefstroom Koekoek performed better in terms of yolk ratio and yolk/albumen than the Lohmann Brown chicken strain. Hanusová et al. [14] also reported significant differences in internal egg quality traits in dual-purpose laying hens of the Oravka and Rhode Island Red breeds. The Oravka breed performed better in terms of albumen and yolk weight. Ukwi et al. [3] found that as the percentage albumen increases, so does the egg weight. Tyasi et al. [22] reported that the breed influenced egg quality traits such as the albumen weight and yolk weight in Potchefstroom Koekoek and Hy-line Silver Brown breeds. The breed influences egg quality of the eggs [14]. Hanusova et al. [23] also examined the parameters of the Rhode Island Red and Oravka eggs and found no differences in albumin content, although there were notable disparities between the two species in terms of egg weight, thickness, Haugh Unit, and yolk colour. Rakonjac et al. [24] compared the eggs produced by Isa Brown hens to ones from New Hampshire chickens, and they concluded that the albumin content varied, while egg weight remained unchanged.

**Table 2:** Mean ± SE of internal egg quality traits affected by genotype.

Egg quality traits	Potchefstroom Koekoek	Lohmann Brown	Probability Value
Yolk weight (g)	15.37^b^ ± 1.09	18.02^a^ ± 1.16	< 0.001
Albumen weight (g)	20.43^b^ ± 2.74	40.69^a^ ± 4.10	< 0.001
Albumen ratio (%)	47.98^b^ ± 5.60	59.71^a^ ± 5.48	< 0.001
Yolk ratio (%)	36.20^a^ ± 2.77	26.52^b^ ± 2.43	< 0.001
Yolk/albumen (%)	76.44^a^ ± 10.20	44.70^b^ ± 5.02	< 0.001

SE: standard error; a, b means in the same row with different superscripts are significantly different (P < 0.05).

### Correlations between egg weight and external egg quality traits

The correlations between egg weight and external egg quality traits of Lohmann Brown and Potchefstroom Koekoek are displayed in Table 3. In Potchefstroom Koekoek, the outcomes displayed that the egg weight had a positive significant (P < 0.05) correlation with egg length and egg width. Furthermore, egg weight was not significantly (P ≥ 0.05) correlated with the shell weight. Lohmann Brown showed that egg weight had a significant positive correlation (P < 0.05) with egg length and width. Lastly, egg weight showed no significant (P ≥ 0.05) correlation with shell weight. The findings of the current study imply that the improvement of the egg length, and egg width may increase the weight of the egg in the Potchefstroom Koekoek and Lohmann Brown layers. The studies conducted by Ukwu et al. [3] in Nigerian Isa Brown egg layer chickens, Dzungwe et al. [25] in French broiler Guinea fowl, and Saroj et al. [18] in indigenous Sakini chickens found that egg length, shell surface area, and egg width were significantly correlated with egg weight.

**Table 3:** Pearson correlation of the egg weight and external egg quality traits with Potchefstroom Koekoek below diagonal and Lohmann Brown above diagonal.

Traits	EW	EL	EWD	SW	SI	USSW	SR
EW (g)		0.41*	0.40*	0.08^ns^	−0.11 ^ns^	−0.37*	−0.49*
EL (mm)	0.29*		0.16^ns^	0.07^ns^	−0.79**	−0.12 ^ns^	−0.17 ^ns^
EWD (mm)	0.44*	0.17^ns^		−0.03 ^ns^	0.47*	−0.22*	−0.26*
SW (g)	0.06^ns^	0.06^ns^	0.34*		−0.08 ^ns^	0.90**	0.83**
SI (%)	−0.09 ^ns^	−0.89**	0.28*	0.08^ns^		−0.02 ^ns^	−0.01 ^ns^
USSW (g/cm^2^)	−0.39*	−0.08 ^ns^	0.11^ns^	0.89**	0.11^ns^		0.99**
SR (%)	−0.50**	−0.12 ^ns^	0.04^ns^	0.83**	0.12^ns^	0.99**	

* Correlation is significant at the 0.05 level, ** Correlation is significant at the 0.01 level, ns: correlation is not significant, EW: egg weight, EL: egg length, EWD: egg width, SW: shell weight, SI: shape index, SSA: shell surface area, USSW: unit shell surface weight, SR: shell ratio.

### Correlations between egg weight and internal egg quality traits

The results of the association between egg weight and internal egg quality traits are shown in Table 4. The results showed that egg weight had a highly significant (P < 0.01) correlation with albumen weight and a significant positive correlation (P < 0.05) with yolk weight in Potchefstroom Koekoek. In Lohmann Brown, egg weight had a positive significant (P < 0.05) correlation with albumen weight. Furthermore, egg weight was not significantly correlated with yolk weight (P ≥ 0.05). The findings imply that improvement of the albumen weight and yolk weight might enhance the egg weight in Potchefstroom Koekoek, while improving the albumen weight may increase the egg weight in Lohmann Brown hens. According to Tyasi et al. [22] and Hlokoe et al. [11], when traits are associated, it suggests that the same gene may be responsible for them. Alkan et al. [4] reported that egg weight was significantly correlated with the albumen ratio and yolk weight in guinea fowls. Ukwu et al. [3] in Nigerian Isa Brown egg layer chickens, Saroj et al. [18] in indigenous Sakini chickens and Tyasi et al. [26] in White Leghorn chickens found a link between egg weight and internal egg quality traits namely, yolk weight, albumen weight and albumen ratio.

**Table 4:** Pearson correlation of the egg weight and internal egg quality traits with Potchefstroom Koekoek below diagonal and Lohmann Brown above diagonal.

Traits	EW	YW	AW	AR	YR	Y/A
EW (g)		0.17^ns^	0.49*	−0.32*	−0.72**	−0.32*
YW (g)	0.46*		0.11^ns^	−0.04^ns^	0.57**	0.49*
AW (g)	0.54**	0.34*		0.67**	−0.33*	−0.81**
AR (%)	−0.04^ns^	0.08^ns^	0.82**		0.24*	−0.62**
YR (%)	−0.58**	0.45*	−0.23*	0.12^ns^		0.61**
Y/A (%)	−0.33*	0.12^ns^	−0.88**	−0.83**	0.44*	

* Correlation is significant at the 0.05 level, ** Correlation is significant at the 0.01 level, ns: correlation is not significant, YW: yolk weight, AW: albumen weight, AR: albumen ratio, YR: yolk ratio, Y/A: yolk/albumen, EV: egg volume.

## Conclusions

The genotype had an effect on external egg quality traits such as egg weight, shell weight, egg length, and egg width in favour of the Lohmann Brown chickens. The breed also influenced internal egg quality traits, such as yolk weight and albumen weight, which favored the Lohmann Brown chickens. Therefore, Potchefstroom Koekoek requires genetic improvement to enhance the egg weight, shell weight, egg length, egg width, yolk weight, and albumen weight. Further studies need to be done to evaluate the egg quality traits of other chicken breeds. The limitation of the study is that the eggs used were collected only at 24 weeks of age, which was not at the hen’s peak production. Therefore, future studies need to be conducted on the eggs collected at the hen’s peak production.
